# Anaesthetic Management of a Patient With LDB3-Associated Myopathy Undergoing Prostate Biopsy Under Total Intravenous Anaesthesia: A Case Report

**DOI:** 10.7759/cureus.102869

**Published:** 2026-02-03

**Authors:** Shashikant Shankar Swami, Mark Campbell, Deepika Rani Basappakokati

**Affiliations:** 1 Anaesthesiology, St Vincents University Hospital, Dublin, IRL; 2 Anesthesia and Critical Care, Tallaght University Hospital, Dublin, IRL; 3 Critical Care Medicine, Mater Misericordiae University Hospital, Dublin, IRL

**Keywords:** cardiomyopathy, ldb3 mutation, myofibrillar myopathy, prostate biopsy, tiva

## Abstract

Mutations in the *LDB3* gene are implicated in myofibrillar myopathy and cardiomyopathy. Consider cardiac conduction defects and the muscle's sensitivity to some anaesthetics when providing anaesthesia in the perioperative period. We present the anaesthetic management of a 76-year-old man with genetically confirmed *LDB3* mutation who presented for prostate biopsy with a background of progressive distal myopathy, mild cardiac involvement, and normal activities of daily living (ADL), undergoing anaesthesia using total intravenous anaesthesia (TIVA) to avoid the use of volatile agents or neuromuscular blockers. In this patient with a history of *LDB3*-associated myopathy, the use of TIVA provided a safe and effective technique of anaesthesia, and reinforced the need for an individualised anaesthetic strategy in neuromuscular disease (NMD).

## Introduction

Inherited neuromuscular diseases (NMDs) comprise a heterogeneous group of disorders characterised by progressive muscle weakness arising from pathology at the level of muscle, peripheral nerve, neuromuscular junction, or motor neuron [1]. Advances in molecular genetics have expanded recognition of late-onset and variably penetrant neuromuscular phenotypes, many of which may remain clinically silent until older adulthood [2]. Within this group, genetically mediated distal myopathies present a particular diagnostic challenge due to overlapping clinical, electrophysiological, and histopathological features [3]. One such condition involves pathogenic variants in the *LDB3* gene, which encodes LIM domain-binding protein 3 (also known as Z-band alternatively spliced PDZ-motif protein, ZASP), a key structural protein involved in sarcomeric organisation and Z-disc stability [4]. *LDB3* mutations have been associated with a spectrum of phenotypes, including distal myopathy, myofibrillar myopathy, and cardiomyopathy [5]. Clinically, affected patients typically present with distal lower limb weakness, foot drop, and slowly progressive functional impairment, while sensory involvement is minimal. Importantly, cardiac manifestations-particularly dilated cardiomyopathy and conduction abnormalities have been described with specific *LDB3* variants, necessitating routine cardiac surveillance.

From an anaesthetic perspective, inherited myopathies such as those associated with* LDB3* mutations have important perioperative implications. These patients may demonstrate altered sensitivity or resistance to neuromuscular blocking agents, unpredictable responses to sedatives and opioids, and an increased risk of postoperative respiratory compromise [6]. Additionally, the potential presence of subclinical cardiomyopathy may increase perioperative cardiovascular risk, even in patients without overt cardiac symptoms. Recognition of the underlying genetic diagnosis is therefore essential to guide anaesthetic planning, intraoperative monitoring, and postoperative care.

We present a case of late-onset, genetically confirmed *LDB3*-associated muscular dystrophy with a distal myopathic phenotype, highlighting the genetic, clinical, and perioperative considerations relevant to anaesthesiologists managing patients with rare inherited neuromuscular disorders.

## Case presentation

A 76-year-old man with a family history of* LDB3*-associated myofibrillar myopathy was booked for an elective transperineal biopsy of the prostate. He was referred for a neurological evaluation due to progressive lower limb weakness. His medical history was notable for hypertension, hypercholesterolaemia, and depression. His regular medications included mirtazapine 30 mg nocte, rosuvastatin 10 mg once daily, and perindopril 8 mg once daily. He reported no known drug allergies.

There was a significant family history of neuromuscular disease. His younger sister had a confirmed diagnosis of *LDB3*-associated muscular dystrophy, while his father had a progressive neuromuscular condition requiring walking aids and wheelchair use in later life, although he was never formally assessed or genetically tested. Several extended family members were also reported to have been clinically affected, suggesting an autosomal dominant inheritance pattern. The patient had been physically active earlier in life, playing football until adolescence and taking up golf in his 30s. His initial symptoms began at the age of 66 years, when he noticed difficulty running across a road. His weakness progressed gradually, with more noticeable deterioration over the preceding two to three years. He reported reduced walking distance, difficulty climbing stairs, frequent tripping, and bilateral foot slap while walking. He did not require walking aids and denied upper limb weakness, sensory symptoms, bulbar symptoms, respiratory complaints, or cardiac symptoms. Functionally, he remained independent in activities of daily living and lived with his wife in an accessible apartment. He was a former smoker with a 35-year smoking history and consumed approximately six units of alcohol per week.

Cardiovascular and respiratory examinations were unremarkable. Cranial nerve examination was normal, with preserved eye movements, facial symmetry, and normal palate and tongue function. Motor examination revealed distal muscle wasting involving the forearms, ankles, and intrinsic foot muscles. Upper limb power was largely preserved, with mild weakness in elbow flexion and extension (Medical Research Council (MRC) grade 4+). Lower limb examination demonstrated mild hip flexion weakness, knee extension graded at MRC 4, ankle dorsiflexion at MRC 4−, plantarflexion at MRC 4, and extensor hallucis longus strength at MRC 4−.

Reflexes were present in the upper limbs and at the knees but absent at the ankles, with flexor plantar responses. Sensory examination showed preserved joint position sense, with reduced vibration sense at the right anterior superior iliac spine and left knee. Gait assessment revealed bilateral foot drop with knee hyperextension during ambulation. He was unable to walk on his heels or toes and required the use of his hands to rise from a supine position. Romberg’s test was negative. Laboratory investigations demonstrated a mildly elevated creatine kinase level of 269 U/L (reference <190 U/L) and elevated troponin T at 22 ng/L (reference <14 ng/L). Vitamin D, vitamin B12, folate, brain natriuretic peptide, renal function, thyroid function tests, liver function tests, glucose, glycated haemoglobin, and homocysteine levels were within normal limits.

Based on the clinical presentation and family history, a distal myopathy with possible mixed neurogenic features was suspected. Genetic testing using a distal myopathy panel, including *LDB3*, was sent, and electromyography was arranged. Anaesthetic planning was therefore undertaken in the context of clinically suspected *LDB3*-associated myopathy and family history, with genetic confirmation obtained subsequently.

Given the recognised association between *LDB3*-associated myopathy and cardiomyopathy, the patient underwent cardiology assessment. He denied exertional chest pain, dyspnoea, syncope, or exercise intolerance and reported only occasional awareness of his heartbeat at night. At cardiology review, his blood pressure was 171/85 mmHg, which he attributed to a white-coat effect, with home blood pressure readings typically in the 120-130 mmHg systolic range. Electrocardiography demonstrated a sinus rhythm with normal axis and intervals. Transthoracic echocardiography revealed aortic sclerosis with mild aortic regurgitation, preserved biventricular systolic function, and normal chamber dimensions. Ambulatory Holter monitoring showed sinus rhythm throughout, with a mean heart rate of 61 beats per minute (range 39-103 bpm) and rare ectopic beats. The cardiology assessment was reassuring, with no evidence of cardiomyopathy or clinically significant arrhythmia at the time of review. A plan was made for repeat electrocardiography and echocardiography in one to two years, with annual neurological follow-up. He was also referred to physiotherapy and occupational therapy. He had never undergone anaesthesia before.

Anaesthetic management

Given the patient’s underlying neuromuscular disease and the potential for altered or unpredictable responses to neuromuscular blocking agents and volatile anaesthetic agents, a total intravenous anaesthesia (TIVA) technique was selected. This approach was chosen to minimise the risk of prolonged neuromuscular weakness, postoperative respiratory compromise, and haemodynamic instability. Standard intraoperative monitoring, in accordance with the Association of Anaesthetists of Great Britain and Ireland (AAGBI) recommendations, was employed, including continuous electrocardiography, pulse oximetry, non-invasive blood pressure monitoring, and capnography. In addition, bispectral index (BIS) monitoring was used to guide the depth of anaesthesia and reduce the risk of intraoperative awareness. General anaesthesia was induced and maintained with propofol and remifentanil via a target-controlled infusion system. Propofol was administered using the Marsh model, and remifentanil using the Minto model. Target plasma concentrations were titrated with propofol around 4-5 µicrograms/mL and remifentanil 3-4 nanograms/mL to maintain BIS values between 40 and 60. Neuromuscular blocking agents and volatile anaesthetic agents were deliberately avoided. Airway management was achieved using a size 4 supraglottic airway device (i-gel), allowing effective ventilation without the need for neuromuscular blockade. The patient was ventilated using pressure support ventilation, with adequate tidal volumes and stable end-tidal carbon dioxide levels maintained throughout. Intraoperative analgesia was provided using a remifentanil target-controlled infusion, titrated to surgical stimulus and haemodynamic response, with small dose long-acting opioids, such as oxycodone 2 milligrams intravenous (IV), along with paracetamol 1 gram IV at the end of the procedure. Antiemetic prophylaxis was employed with ondansetron 4 milligrams IV, given the use of total intravenous anaesthesia and the aim of facilitating rapid recovery and same-day discharge. Intraoperative fluid management consisted of a restricted crystalloid strategy, with approximately 500 millilitres of Ringer's lactate solution to maintain euvolaemia and stable haemodynamics. Core temperature was monitored, and normothermia was maintained using a forced-air warming blanket. 

The surgical procedure lasted approximately 45 minutes. Intraoperative haemodynamics remained stable, with no episodes of hypotension, bradycardia, or arrhythmia. Emergence from anaesthesia was smooth, with prompt recovery of spontaneous ventilation and consciousness. Time to eye opening and obeying commands was around 6 to 7 minutes after stopping TIVA. Oxygen requirement in post anesthesia care unit (PACU) was on nasal prongs with a 2 L/min flow with a target oxygen saturation (SpO₂) of more than 94%. Patient was comfortable with a pain score of 2 out of 10, and no rescue opioid analgesia was required. No postoperative nausea or vomiting was reported in the post-anaesthesia care unit. Patient stayed in PACU for 60 minutes. Postoperative recovery was uneventful, with no evidence of residual sedation, respiratory compromise, or neuromuscular weakness. The patient met discharge criteria without delay and was discharged home on the same day from the day ward. 

## Discussion

Anaesthetic considerations with *LDB3 *mutations relate to the myopathy and heart involvement. Malignant hyperthermia has not been described with *LDB3*, but caution may be warranted, as there is overlap with other myopathy types. TIVA avoids known triggers while providing complete control of the depth of anaesthesia. Airway management was achieved without neuromuscular blockade using a supraglottic airway device, supporting rapid emergence and reducing the risk of postoperative respiratory compromise. This case stresses the value of individualised anaesthesia care plans and preoperative multidisciplinary assessment.

A pediatric case series on myofibrillar myopathy has warned against the use of volatile anaesthetics and depolarising neuromuscular blockers [6], which aligns with our management, but neuraxial anaesthesia has been used in similar adults for obstetric surgery [7]. While the association of MH with LDB3 is not established, given phenotypic similarities to other myopathies associated with MH, avoidance of volatile anaesthetic agents and the administration of TIVA to prevent exposure to a known trigger appear wise [8,9]. The risk of other anaesthetic side effects, such as non-depolarising neuromuscular blockade, should also be minimised. In this case, volatile anaesthetics and depolarising neuromuscular blockers were avoided as a precautionary, risk-avoidance strategy given the broader uncertainties that can accompany inherited myopathies, rather than because of a proven association with malignant hyperthermia.

*LDB3* mutations affect the Z-disc of striated muscle, and cause myofibrillar myopathy, dilated cardiomyopathy (DCM) and left ventricular non-compaction (LVNC). Conditions associated with *LDB3* have different implications for anaesthesia due to drug sensitivity in myopathy and cardiac involvement. Even in cases with asymptomatic cardiac involvement, OrphanAnaesthesia guidelines advise maintaining hemodynamic stability in patients with LVNC and avoiding triggers known to induce arrhythmias [10].

Anaesthetic drugs should be of particular concern in myopathies. There is evidence that opioids and sedatives cause increased sensitivity, and dosages should be adjusted to the individual [11]. Myopathy patients can become rapidly fatigued and have a decreased pulmonary reserve, increasing the risk of perioperative complications. However, the risk can be reduced with careful titration and monitoring of anaesthetic agents [12]. Induction and maintenance anaesthetic requirements may be altered by multisystem disease involving neuromuscular impairment and hepatic and renal comorbidities, which may alter drug metabolism [13]. In this report, even minor and apparently non-intrusive procedures in patients with muscular dystrophy were reported to be associated with prolonged recovery time and postoperative ventilation requirements [14].

TIVA has been shown to be a safe and effective anaesthetic alternative for patients with a variety of myopathies when avoidance of volatile agents and neuromuscular blockers is required [15]. Target-controlled infusion systems rely on population pharmacokinetic models and may demonstrate clinically relevant prediction error in individual patients. The Marsh propofol and Minto remifentanil models have been shown to inaccurately predict plasma and effect-site concentrations, with substantial inter-individual variability, particularly in older patients and those with altered physiology [16]. Accordingly, in this case, the TCI models were used as guiding frameworks, with anaesthetic depth titrated primarily to clinical response and bispectral index monitoring, rather than reliance on model-predicted concentrations alone. Plasma-site targeting offers greater haemodynamic stability by avoiding rapid surges in effect-site concentration that can occur with effect-site targeting, thereby reducing abrupt vasodilation and myocardial depression. This allows more controlled titration and may be advantageous in patients with potential cardiac involvement. TIVA (as used in this case) and avoiding the use of muscle relaxants are recommended in this population and have contributed to the stable perioperative course.

Transperineal prostate biopsy can often be performed under local anaesthesia, neuraxial anaesthesia, or monitored anaesthesia care, and it does not inherently require neuromuscular blockade. In progressive myopathy, regional anaesthesia carries a theoretical risk of unpredictable block effects and prolonged motor weakness. In our setting, general anaesthesia without neuromuscular blockade was selected due to institutional practice, patient preference and positioning tolerance, with the goal of providing predictable conditions while minimising exposure to volatile agents and neuromuscular blockers in a patient with an inherited myopathy. We acknowledge that less invasive techniques would be reasonable alternatives and should be considered on an individual basis. Although transperineal prostate biopsy is a minor procedure, total intravenous anaesthesia represents standard practice at our institution rather than a complex technique and is routinely delivered by consultants with dedicated training and experience in TIVA.

Given the rarity of genetically confirmed *LDB3*-associated myopathy and the limited anaesthetic literature, this case represents a unique clinical scenario requiring an individualised and precautionary anaesthetic approach.

## Conclusions

This case describes the perioperative management of a patient with genetically confirmed *LDB3*-associated neuromuscular disease undergoing anaesthesia. Given the presence of progressive muscle weakness and the potential for cardiac involvement, careful preoperative assessment, including cardiology review, was central to perioperative planning. Anaesthesia was delivered using a total intravenous technique without neuromuscular blocking agents, allowing effective airway management and stable intraoperative conditions. The patient experienced an uncomplicated recovery with no evidence of respiratory compromise or residual weakness. This tailored approach contributed to a smooth perioperative course and same-day discharge.
